# Estimating latent individual demographic heterogeneity using structural equation models

**DOI:** 10.1002/ecy.70161

**Published:** 2025-08-06

**Authors:** Thomas V. Riecke, Rémi Fay, Johann Hegelbach, Pierre‐Alain Ravussin, Daniel Arrigo, Michael Schaub

**Affiliations:** ^1^ Swiss Ornithological Institute Sempach Switzerland; ^2^ Wildlife Biology Program, Department of Ecosystem and Conservation Science University of Montana Missoula Montana USA; ^3^ Laboratoire de Biométrie et Biologie Évolutive Unité Mixte de Recherche (UMR) 5558, Université Claude Bernard Lyon 1, CNRS, VetAgro Sup Villeurbanne France; ^4^ Department of Evolutionary Biology and Environmental Studies University of Zurich Zurich Switzerland; ^5^ Baulmes Switzerland; ^6^ Nidau Switzerland

**Keywords:** *Cinclus cinclus*, demography, *Ficedula hypoleuca*, fitness, individual heterogeneity, residual reproductive value, senescence, structural equation model

## Abstract

Understanding the drivers of fitness is a key goal of population and evolutionary ecology. However, measuring individual variation in demographic components in imperfectly observed populations of wild organisms is extremely challenging. Recent research has demonstrated that estimates of fixed individual variation in Bernoulli variables (e.g., survival and breeding propensity) are often unreliable in the face of imperfect detection and small sample sizes. Thus, we demonstrate the use of structural equation modeling approaches to simultaneously estimate latent variation in demographic performance and link said variation to individual demographic components. We demonstrate the use of this approach with 30+ year capture–recapture datasets collected on two passerine species (white‐throated dipper, *Cinclus cinclus*, and pied flycatcher, *Ficedula hypoleuca*) and simultaneously estimate latent variation in individual quality and age‐specific variation in demographic components. We found senescent decline in survival and fecundity in both species and demonstrated strong among‐individual heterogeneity in demographic performance. Notably, the residual reproductive value of older individuals of higher quality was greater than younger individuals of reduced and average quality. We demonstrate that this approach may be useful in a variety of situations, discuss its limitations, and make suggestions for future research.

## INTRODUCTION

Understanding age‐specific variation and fixed or static individual heterogeneity in demographic parameters such as survival and fecundity (Fay, Authier, et al., 2022; Forsythe et al., 2021; Gimenez et al., 2018; Hamel et al., 2018; Monaghan et al., 2008; Nussey et al., 2008) is critical for an understanding of individual fitness (Newton, 1989; Stearns, 1992). The accurate estimation of age‐specific changes in demographic parameters is dependent on the accurate estimation of latent individual heterogeneity, as average patterns at the population level often do not match within individual rates of decline due to selective disappearance (Nussey et al., 2008; Vaupel & Yashin, 1985). For example, if survival and fecundity are positively correlated within individuals (Cam et al., 2002; Fay, Hamel, et al., 2022), individuals with higher latent fecundity will be overrepresented in later age classes as less fecund individuals who also have lower survival probabilities will disappear from the population early in life (Vedder & Bouwhuis, 2018).

Previous approaches for estimating age‐specific demographic parameters have often relied on specifying individual random effects or “frailties” (e.g., Marzolin et al., 2011; Vaupel et al., 1979) to control for latent, or unmeasured, fixed among‐individual variation in demographic performance. When more than one demographic component is estimated, bivariate or multivariate normal distributions are often used to separately assess fixed individual variation in each demographic component around population means, and simultaneously estimate correlations among demographic components as well as linear (Cam et al., 2002), nonlinear (Marzolin et al., 2011), or categorical (Fay et al., 2021) effects of age. Critically, recent research has revealed that accurately estimating among‐individual variance in imperfectly observed Bernoulli variables can be challenging (Fay, Authier, et al., 2022), even given substantial sample sizes. Further, inappropriate Bayesian priors can lead to overestimates of variance in individual heterogeneity and underestimates of correlations among demographic components (Fay, Authier, et al., 2022; Riecke et al., 2019). Beyond these obvious problems, these challenges can also lead to inaccurate estimates of the shape and rate of senescent decline or the effects of other covariates on different demographic components. Thus, developing models that can accurately estimate individual heterogeneity and relationships among demographic components will be critical for improving estimates of individual fitness in imperfectly observed populations of organisms.

Causal, or structural equation, models have been rapidly growing in popularity to address complex ecological problems (e.g., Frauendorf et al., 2021; Grace et al., 2010) and previous work on the estimation of individual “quality” has suggested the use of causal models (Wilson & Nussey, 2010). However, structural equation models have not yet been applied to estimate individual heterogeneity in demographic performance in capture–recapture frameworks. In this paper, we demonstrate the use of structural equation models (Cubaynes et al., 2012; Grace et al., 2010; Pearl, 2000) and longitudinal capture–recapture and fecundity data to simultaneously estimate fixed latent individual heterogeneity in demographic performance, as well as linkages between this heterogeneity and survival and reproduction. Thus, rather than estimating an individual random effect for every measured demographic component and estimating correlations among demographic components (Cam et al., 2002; Fay, Hamel, et al., 2022), using structural equation models we estimate a single latent “quality” for each individual, and relationships between that latent quality and demographic components. This reduces the number of parameters researchers must estimate while retaining a similar underlying model structure. We apply these models to data collected on pied flycatchers (*Ficedula hypoleuca*; 1980–2016) and white‐throated dippers (*Cinclus cinclus*; 1989–2018) breeding in Switzerland, and estimate age‐specific demographic rates, latent individual heterogeneity in demographic performance (Cubaynes et al., 2012), and relationships between latent quality and demographic components for each species. We then calculate the apparent (i.e., local) residual reproductive value of individuals of differing latent demographic qualities at different age classes and discuss the implications of this new modeling tool for estimating latent variation in individual quality in imperfectly observed populations of wild organisms.

## METHODS

Pied flycatcher populations were monitored at Baulmes (1980–2016; 46.7894° N, 6.5225° E) and Corcelles‐près‐Concise/Onnens, Switzerland (1987–2016; 46.8400° N, 6.7070° E). Juveniles were uniquely marked as nestlings, and adult females were marked and recaptured at nest boxes during egg laying, incubation, or brood rearing. See Fay et al. (2021) for further details regarding pied flycatcher data collection. Dipper populations were monitored along three streams, Sihl, Werlenbach, and Küsnachterbach, near Zurich (1989–2018; 47.3777° N, 8.5369° E). Nestlings were uniquely marked with rings, and later recaptured and marked with a unique combination of plastic bands. Adults and recruiting offspring were resighted and recaptured throughout the year. See Becker et al. (2015) for further details regarding dipper data collection. The data (Y) were then summarized in a three‐dimensional array where rows represent individuals (i), columns represent years (t), the first “layer” of the array ($y_{i,t,1}$) consists of the mark‐reencounter data (1: observed, 0: not observed), and the second “layer” of the array ($y_{i,t,2}$) consists of the number of local recruits produced during a breeding season (t) by each individual (i). Notably, both juveniles and adults can disperse outside of the bounds of both study areas. Thus, the results we present here are also a function of both natal and adult dispersal, and presumably underestimate both true survival and reproductive success.

We used the same model for the dipper and flycatcher datasets, and included individuals of both known and unknown age. To include individuals of unknown age, we estimated the age of each individual i during its first observed breeding attempt ($f_{i}$) given an age distribution (π), $a_{i,f_{i}}˜\text{categorical}\left(\pi \right)$. We allowed for first observed breeding from one to seven years of age, where we specified vague priors for each age class (k), $\pi_{k}^{*}˜\text{gamma}\left(1,1\right)$, and then derived age‐specific probabilities of first encounter, $\pi_{k}=\frac{\pi_{k}^{*}}{\sum \pi^{*}}$. Individuals aged between years, where $a_{i,t+1}=a_{i,t}+1$ following each individual's initial encounter.

We modeled the observation of each individual during each year ($y_{i,t,1}$) as a function of individual latent state ($z_{i,t}$; 1 = alive, 0 = dead) and year‐specific detection probability ($p_{t}$), and we modeled each ringed individual's latent state ($z_{i,t}$) as a function of an individual (i) and year‐specific (t) survival probability ($\phi_{i,t}$),

(1) $$\begin{array}{l} y_{i,t,1}˜\left\{\begin{array}{ll} \text{Bernoulli}\left(p_{t}\right), & z_{i,t}=1\\ 0, & z_{i,t}=0 \end{array}\right.,\\ z_{i,t}˜\left\{\begin{array}{ll} \text{Bernoulli}\left(\phi_{i,t-1}\right), & z_{i,t-1}=1\\ 0, & z_{i,t-1}=0 \end{array}\right.. \end{array}$$

We first estimated latent heterogeneity in quality (η) among individuals for each species with a vague prior for variance:

(2) $$\begin{array}{l} \eta_{i}˜\text{normal}\left(0,\sigma_{\eta }^{2}\right),\\ \sigma_{\eta }˜\text{gamma}\left(1,1\right). \end{array}$$

For dippers, we then estimated apparent survival probability ($\phi_{i,t}$) as a function of each individual's age‐specific mortality hazard rate (Ergon et al., 2018). Mortality hazard rate was modelled with an effect ($\gamma_{\phi }$) of latent individual quality ($\eta_{i}$), an effect of age, and random temporal variation in mortality hazard rate ($\kappa_{t}$):
(3)

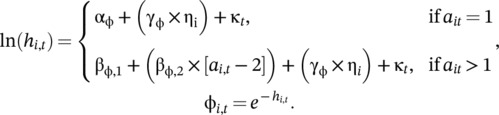




We used a similar but simpler model for the flycatcher data, where mortality hazard rate increased linearly as a function of age, $\ln\left(h_{i,t}\right)=\beta_{\phi ,1}+\left(\beta_{\phi ,2}\times \left[a_{i,t}-1\right]\right)+\left(\gamma_{\phi }\times \eta_{i}\right)+\kappa_{t}$. We specified vague priors for the effect of age, $\beta_{\phi }˜\text{normal}\left(0,100\right)$, the effect of individual heterogeneity, $\gamma_{\phi }˜\text{normal}\left(0,100\right)$, random year effects, $\kappa_{t}˜\text{normal}\left(0,\sigma_{\kappa }^{2}\right)$, and variation among years, $\sigma_{\kappa }˜\text{gamma}\left(1,1\right)$, for both models.

We modeled year‐specific detection probability ($p_{t}$) of marked individuals as random temporal variation ($\sigma_{p}^{2}$) around a mean ($\mu_{p}$), $\text{logit}\left(p_{t}\right)˜\text{normal}\left(\mu_{p},\sigma_{p}^{2}\right)$. We specified vague priors; $\mu_{p}˜\text{normal}\left(0,10\right)$, and $\sigma_{p}˜\text{gamma}\left(1,1\right)$. Given that flycatcher data were collected at two sites by two observers, we specified site‐specific means and temporal variances for flycatcher detection probability at each site. We did not include site‐specific variation in the dipper data, although they originate from three streams, because the resighting effort was equivalent at all three streams and collected by the same observer.

Similarly, we modeled the expected number of recruiting offspring from each individual's breeding attempt or attempts during each year ($y_{i,t,2}$) as a function of an intercept equivalent to fecundity at age one ($\alpha_{\xi }$), a quadratic change in recruitment ($\beta_{\xi }$) as a function of age, as similar patterns have been observed in both species in this study system (Fay et al., 2021; Riecke et al., 2023), an effect ($\gamma_{\xi }$) of latent heterogeneity in quality ($\eta_{i}$), and random temporal variation in fecundity ($\omega_{t}$):
(4)

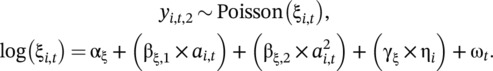




Thus, $\eta_{i}$ serves as a “latent variable” as defined in structural equation modeling frameworks (Grace et al., 2010) representing individual variation in demographic performance. We fixed $\gamma_{\xi }=1$ for parameter identifiability as is standard in structural equation modeling (Cubaynes et al., 2012; Grace et al., 2010), and specified vague priors for other regression parameters; $\beta_{\xi }˜\text{normal}\left(0,100\right)$, $\omega_{t}˜\text{normal}\left(0,\sigma_{\omega }^{2}\right)$, and $\sigma_{\omega }˜\text{gamma}\left(1,1\right)$.

We conducted analyses in R (R Core Team, 2020) and JAGS (Plummer, 2003) using the jagsUI package (Kellner, 2016). For each species, we sampled three MCMC chains for 250,000 iterations, discarding the first 100,000 iterations and retaining every 10th saved iteration. We visually inspected trace plots for convergence (Kéry & Schaub, 2012). In the following text, tables, and figures, we report medians of posterior distributions, 95% Bayesian credible intervals (CIs), and υ, the proportion of the posterior distribution on the same side of zero as the mean.

We used Bayesian posterior distributions from the previously described model parameters to estimate apparent residual reproductive value of each age class ($R_{a}$) across ranges of latent individual quality ($\eta_{i}$) for each species (Bouwhuis et al., 2012; Newton & Rothery, 1997). At the beginning of each reproductive attempt, we summed the expected number of recruits produced during that age‐ and heterogeneity specific attempt, as well as the residual expectation of subsequent attempts until age at last reproduction (ALR = 7) given survival to each breeding season:

(5) $$R_{a}=\sum_{k=a}^{\mathrm{ALR}}\;\frac{l_{k}}{l_{a}}\xi_{k}.$$

Here, we define $\frac{l_{k}}{l_{a}}$ as the probability of survival from a to k given previous survival to a (e.g., survival from the fourth to sixth breeding season is equal to, $\frac{l_{6}}{l_{4}}=\phi_{4}\times \phi_{5}$), and $\xi_{k}$ is the expected number of recruits produced during a reproductive attempt at age k. We repeated this calculation across gradients of estimated individual heterogeneity in latent quality ($\eta_{i}=\left[-\sigma_{\eta },0,\sigma_{\eta }\right]$) for both species. Data and code for all analyses are archived at Dryad: https://doi.org/10.5061/dryad.jm63xsjp5.

### Data simulation and analysis

To assess the efficacy of the approach we describe herein, we generated simulated data and compared the performance of both multivariate normal (MVN) and structural equation (SEM) capture‐mark‐recapture models. We first simulated correlated heterogeneity in survival and reproduction using a bivariate normal distribution,
(6)

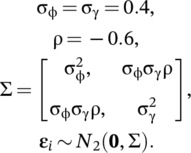




Thus, we simulated a negative correlation between mortality hazard rate and reproductive rate (i.e., a positive relationship between survival and reproductive rate). We then simulated individual survival probabilities (ϕ) given mortality hazard rates (h; Ergon et al., 2018) and individual reproductive rate (γ) as a function of correlated individual random effects and means (μ):
(7)

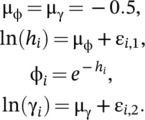




To mimic our study populations, we simulated the release of 25 individuals per year for 20 years, and simulated individual latent states and reproductive success given individual survival probability and reproductive rate (Schaub & Kéry, 2021). We then generated capture histories given a constant detection probability (p=0.8).

We fit two models to each dataset, the data generating model (i.e., bivariate normal) and a structural equation model. We assigned vague priors for the bivariate normal model:
(8)

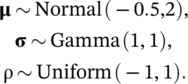




For the structural equation model, we estimated an individual heterogeneity in demographic performance, $\eta ˜\text{Normal}\left(0,\sigma_{\eta }^{2}\right)$, with a vague prior on the variance, $\sigma_{\eta }˜\text{Gamma}\left(1,1\right)$. We then parameterized survival and reproductive rate as a function of the individual heterogeneity:

(9) $$\begin{array}{c} \ln\left(h_{i}\right)=\mu_{\phi }+\lambda \times \eta_{i},\\ \;\phi_{i}=e^{-h_{i}},\\ \;\ln\left(\gamma_{i}\right)=\mu_{\gamma }+\eta_{i},\\ \;\mu ˜\text{Normal}\left(-0.5,2\right),\\ \;\lambda ˜\text{Normal}\left(0,2.5\right). \end{array}$$

We used Stan (Stan Development Team, 2024) in R (R Core Team, 2024) via rstan (Stan Development Team, 2025) for computational efficiency. We ran a total of 1000 simulations, sampled four HMC chains for 10,000 iterations, discarding the first 5000 iterations and retaining every 5th saved iteration. We assessed convergence via $\hat{R}<1.05$ (Vehtari et al., 2021), and present mean signed deviation ($\mathrm{MSD}\left(\hat{\theta }\right)=\frac{1}{n}\sum_{i=1}^{n}\;{\hat{\theta }}_{i}-\theta$), and coverage, or the proportion of simulations in which the 95% CIs contain the data generating value, in the results. Data and code for all analyses are archived at Dryad: https://doi.org/10.5061/dryad.jm63xsjp5.

## RESULTS

We monitored 1001 breeding attempts by 588 uniquely marked female pied flycatchers (290 of unknown age) that produced 476 recruits, and 1322 breeding attempts by 674 uniquely marked female dippers (354 of unknown age) that produced 865 recruits.

### Latent fitness variation and age‐specific demographic rates

**FIGURE 1:**
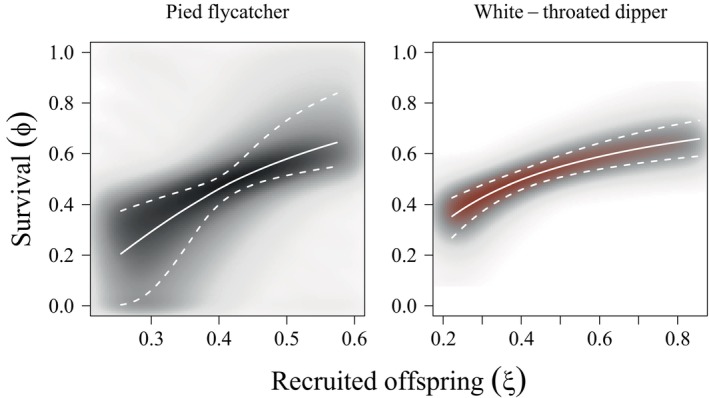
Posterior distributions (shaded gradients), medians (solid lines), and 90% Bayesian credible intervals (dashed lines) for joint estimates of apparent survival (ϕ) and the mean number of local recruits produced per breeding season (ξ) of one‐year‐old European pied flycatcher (left; *Ficedula hypoleuca*) and white‐throated dipper (right; *Cinclus cinclus*) females breeding in Switzerland. The density of the shading corresponds to the density of the posterior distribution.

**FIGURE 2:**
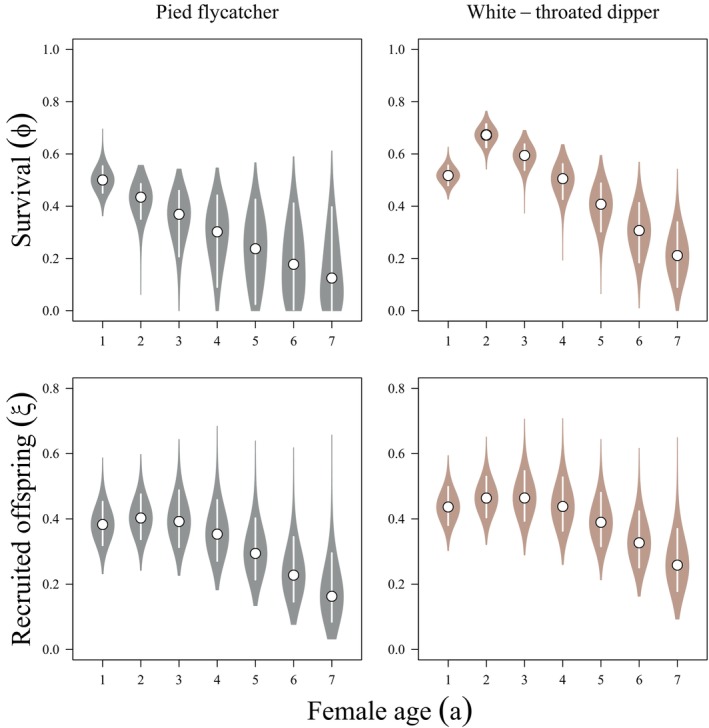
Posterior distributions (violin plots), medians (points), and 90% Bayesian credible intervals (lines) of estimates of apparent survival (ϕ; top) and the mean number of locally recruited offspring per breeding season (ξ; bottom) for European pied flycatcher (*Ficedula hypoleuca*; left) and white‐throated dipper (*Cinclus cinclus*; right) females breeding in Switzerland.

We estimated latent heterogeneity in flycatcher ($\sigma_{\eta }=0.405$; 95% CI: 0.181, 0.608) and dipper ($\sigma_{\eta }=0.672$; 95% CI: 0.554, 0.799) demographic performance. We estimated strong negative relationships between latent quality (η) and mortality hazard rate for both flycatchers ($\gamma_{\phi }=-1.573$; 95% CI: −5.193, −0.538) and dippers ($\gamma_{\phi }=-0.675$; 95% CI: −1.106, −0.353). As latent quality was scaled to individual variation in fecundity ($\gamma_{\xi }=1$), individuals with increased fecundity experience reduced mortality (Figure 1). We estimated increases in mortality hazard rate with increasing age for both flycatchers ($\beta_{\phi }=0.184$; 95% CI: 0.018, 0.504) and dippers ($\beta_{\phi }=0.274$; 95% CI: 0.179, 0.379) following an decline in mortality hazard rate from year one to year two for dippers (Figure 2). Similarly, the annual number of offspring recruited declined with age for both flycatchers ($\beta_{\xi ,2}=-0.038$; 95% CI: −0.083, 0.002; $\upsilon_{\beta_{\xi ,2}}=0.969$) and dippers ($\beta_{\xi ,2}=-0.029$; 95% CI: −0.057, −0.003; $\upsilon_{\beta_{\xi ,2}}=0.987$) females (Figure 2).

### Residual reproductive value

**FIGURE 3:**
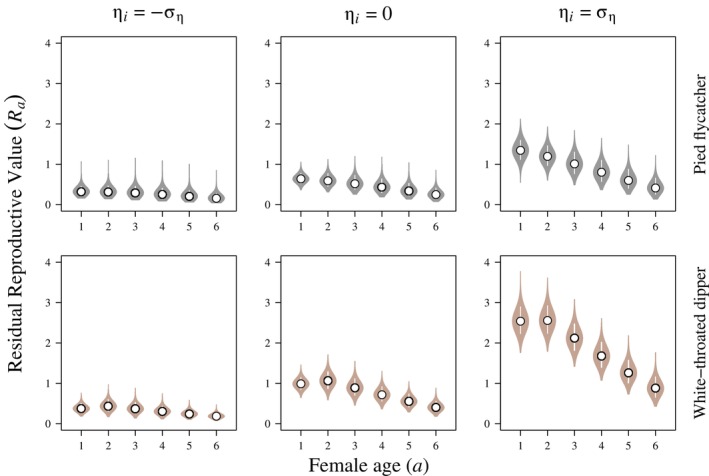
Posterior distributions of apparent (i.e., local) age‐specific residual reproductive value ($R_{a}$; i.e., expected additional lifetime recruited offspring) of one through six‐year‐old European pied flycatcher (*Ficedula hypoleuca*; top) and white‐throated dipper (*Cinclus cinclus*; bottom) females breeding in Switzerland as a function of latent individual variation in demographic performance or “quality” ($\eta_{i}$). The white points represent medians and white lines denote 90% Bayesian credible intervals.

Given estimated latent heterogeneity, there was substantial individual variation in residual reproductive value for both flycatchers and dippers (Figure 3). Individual flycatchers with elevated latent quality ($+1\sigma_{\varepsilon }$) had 2.1 times the residual reproductive value of average ($\sigma_{\varepsilon }=0$) flycatchers, and 4.2 times the residual reproductive value of flycatchers with reduced latent quality ($-1\sigma_{\varepsilon }$) at age one. Similarly, individual dippers with increased latent quality had 2.6 times the residual reproductive value of average dippers, and 6.7 times the residual reproductive value of dippers with reduced latent quality at age one. As individuals of both species aged, senescent increase in mortality hazard rates and fewer remaining breeding attempts reduced this difference. At age six, flycatchers with elevated latent quality had 1.6 and 2.6 times the residual reproductive value of average and low‐quality flycatchers, and high‐quality dippers had 2.2 and 4.7 times the residual reproductive value of average and low‐quality dippers (Figure 3).

### Simulation results

**FIGURE 4:**
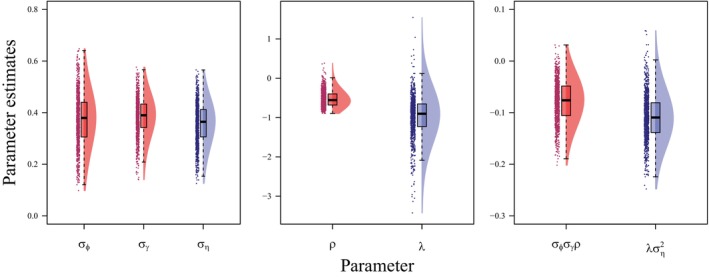
Modified raincloud plots (Allen et al., 2021) of medians of posterior distributions for estimates of SD (σ; left), correlation (ρ) or regression (λ; center), and “covariance” (right) parameters from bivariate normal (red) and structural equation (blue) models estimating correlated individual heterogeneity in survival (ϕ) and fecundity (γ) from 1000 simulations of capture‐mark‐recapture data.

Both models achieved convergence for 998 of the 1000 simulated datasets. Estimates of correlations from bivariate normal models were slightly biased toward zero (MSD = 0.075; Figure 4), while coverage was adequate (0.994). Estimates of SDs from bivariate normal models were constant and coverage was adequate for both mortality hazard rate ($\sigma_{\phi }$; MSD = −0.026, coverage = 0.951) and reproductive rate ($\sigma_{\gamma }$; MSD = −0.017, coverage = 0.944). We interpreted the proportion of simulations in which the upper confidence interval was less than zero for estimates of correlations (ρ; bivariate normal) and regression parameters (λ; structural equation models) as evidence for a negative relationship between mortality hazard and fecundity rates. Structural equation models recovered evidence for a negative relationship between mortality hazard and fecundity rates in more than three‐quarters of simulations (0.767), while bivariate normal models recovered evidence in slightly less than half of simulations (0.498), despite using a bivariate normal model to generate the data (Figure 4).

## DISCUSSION

We observed strong associations between apparent (i.e., local) offspring recruitment and mortality in both dipper and flycatcher females (Figure 1). We also observed strong senescence in survival: survival probabilities of two‐year‐old dippers and flycatchers were 3.2 and 2.9 times greater than seven‐year‐old individuals, respectively. Fecundity declined across the lifetime of individuals in both species (Figure 2). Accordingly, residual reproductive value declined as individuals aged. Notably, residual reproductive value declined more strongly with age in individuals with increased latent quality, as the survival probability of lower quality individuals was prohibitively low, reducing the value of future reproductive attempts beyond the current breeding season. Further, the residual reproductive value of older individuals with higher latent quality was greater than the residual reproductive value of younger individuals with lower latent quality (Figure 3).

### Senescence, heterogeneity, and lifetime fitness

Senescent decline is ubiquitous in vertebrate populations (Nussey et al., 2008). Similar to other studies (e.g., Cam et al., 2002), we observed substantial heterogeneity in the demographic performance of individuals, suggesting selective disappearance of less fecund individuals over time (Vaupel & Yashin, 1985). After controlling for this heterogeneity using structural equation modeling approaches, we observed senescent decline in both survival and fecundity in dipper and flycatcher populations (Figure 2). These findings have important implications for the estimation of lifetime fitness and population trends, as they may allow for improved assessments of latent variation in fitness among individuals.

The determinants of lifetime reproductive success are complex (Newton, 1989; Stearns, 1992). For example, environmental conditions during growth and development may affect the fixed condition of individuals (Forsythe et al., 2021) and early‐life decisions can affect late‐life patterns of senescent decline (Aubry et al., 2009). In this study, we demonstrate strong variation in expected residual reproductive value as a function of within‐individual associations between demographic components (Figure 1), while simultaneously estimating temporal variation in the same components. Future research might address the genetic, environmental, and life‐history drivers that lead to individual heterogeneity in demographic components (e.g., Aubry et al., 2009).

### On the estimation of individual heterogeneity, age structure, and parameter constancy

Critically, accurate age‐specific estimates of demographic rates are conditional on the accurate estimation of individual heterogeneity in demographic components (Cam et al., 2002; Nussey et al., 2008). Recent research (Fay, Authier, et al., 2022) has conclusively demonstrated that estimates of individual heterogeneity for Bernoulli variables can be poorly estimated when sample sizes are low. Further, the use of inappropriate priors can lead to inaccurate estimates (Fay, Authier, et al., 2022; Riecke et al., 2019). Sample sizes for both species in the study were relatively small, particularly when compared to long‐term studies of some other species, such as colonial seabirds (e.g., Cam et al., 2002; Fay et al., 2018). However, the use of structural equation models in this study allowed for the estimation of heterogeneity in latent quality rather than estimating a heterogeneity or individual random effect for each individual demographic component. This reduces the number of parameters relative to multivariate normal approaches and thus reduces the challenges associated with estimating variances of Bernoulli variables and correlations among demographic parameters.

### Data simulation

Similar to Fay, Authier, et al. (2022), we observed that bivariate normal models underestimate correlations (ρ) when sample sizes are low. Our simple simulation clearly demonstrates that structural equation models can more effectively recover evidence of relationships among demographic parameters than bivariate normal models, even when bivariate normal models are used to generate the data. Thus, we suggest that these models may be quite useful for examining within‐individual relationships among demographic parameters, particularly in situations where available data may constrain the use of bi‐ or multi‐variate normal distribution parameterizations. Future research should further explore the behavior and performance of these models as a function of the number of marked individuals, study duration, and priors.

### Implications for future research

The structural equation modeling approach we describe in this paper requires fewer parameters than approaches based on multivariate normal distributions (Fay, Authier, et al., 2022; Riecke et al., 2019), allowing for the estimation of relationships between a single latent heterogeneity and individual demographic parameters. Critically, we note that the sources of bias described in the preceding paragraphs are not unique to the model we describe here, as they will also affect parameter constancy in other existing model structures, such as models relying on multivariate normal distributions. Although longitudinal studies of populations with known‐age individuals and near‐perfect observation of marked individuals (e.g., Cam et al., 2002) will always provide enhanced resolution of senescence and individual heterogeneity (Cam et al., 2016), the model we describe herein has the potential to directly estimate latent variation in individual demographic performance and link said variation to multiple demographic components. We note continued challenges related to the estimation of heterogeneity in Bernoulli processes (Fay, Authier, et al., 2022). We suggest that the method described herein has great utility and might easily be expanded to incorporate multiple components of fecundity (e.g., age at first reproduction, breeding propensity). Similarly, latent variables may also be modeled as a function of covariates (Grace et al., 2010), allowing researchers to examine the drivers of variation in demographic performance across multiple traits. Thus, the method we describe herein is a simplified extension of previously described approaches (Link et al., 2002; Wilson & Nussey, 2010) that may allow for more accurate estimation of linkages among demographic components. In particular, we are greatly excited by recent developments in collective data storage of long‐term capture–recapture studies (e.g., Culina et al., 2021; Salguero‐Gómez et al., 2016), and note the potential to address similar questions with greater precision at broader spatiotemporal and phylogenetic (Abadi et al., 2014) scales in the future.

## CONFLICT OF INTEREST STATEMENT

The authors declare no conflicts of interest.

## Data Availability

Data and code (Riecke et al., 2025) are available in Dryad at https://doi.org/10.5061/dryad.jm63xsjp5.

## References

[ecy70161-bib-0001] Abadi, F. , C. Barbraud , D. Besson , J. Bried , P.‐A. Crochet , K. Delord , J. Forcada , et al. 2014. “Importance of Accounting for Phylogenetic Dependence in Multi‐Species Mark–Recapture Studies.” Ecological Modelling 273: 236–241.

[ecy70161-bib-0002] Allen, M. , D. Poggiali , K. Whitaker , T. R. Marshall , J. van Langen , and R. A. Kievit . 2021. “Raincloud Plots: A Multi‐Platform Tool for Robust Data Visualization.” Wellcome Open Research 4: 63. 31069261 10.12688/wellcomeopenres.15191.1 PMC6480976

[ecy70161-bib-0003] Aubry, L. M. , D. N. Koons , J.‐Y. Monnat , and E. Cam . 2009. “Consequences of Recruitment Decisions and Heterogeneity on Age‐Specific Breeding Success in a Long‐Lived Seabird.” Ecology 90: 2491–2502. 19769127 10.1890/08-1475.1

[ecy70161-bib-0004] Becker, P. J. , S. Reichert , S. Zahn , J. Hegelbach , S. Massemin , L. F. Keller , E. Postma , and F. Criscuolo . 2015. “Mother–Offspring and Nest‐Mate Resemblance but no Heritability in Early‐Life Telomere Length in White‐Throated Dippers.” Proceedings of the Royal Society B: Biological Sciences 282: 20142924. 10.1098/rspb.2014.2924 PMC4424638 25904662

[ecy70161-bib-0005] Bouwhuis, S. , R. Choquet , B. C. Sheldon , and S. Verhulst . 2012. “The Forms and Fitness Cost of Senescence: Age‐Specific Recapture, Survival, Reproduction, and Reproductive Value in a Wild Bird Population.” The American Naturalist 179: E15–E27. 10.1086/663194 22173469

[ecy70161-bib-0006] Cam, E. , L. M. Aubry , and M. Authier . 2016. “The Conundrum of Heterogeneities in Life History Studies.” Trends in Ecology & Evolution 31: 872–886. 27665020 10.1016/j.tree.2016.08.002

[ecy70161-bib-0007] Cam, E. , W. A. Link , E. G. Cooch , J.‐Y. Monnat , and E. Danchin . 2002. “Individual Covariation in Life‐History Traits: Seeing the Trees despite the Forest.” The American Naturalist 159: 96–105. 10.1086/324126 18707403

[ecy70161-bib-0008] Cubaynes, S. , C. Doutrelant , A. Grégoire , P. Perret , B. Faivre , and O. Gimenez . 2012. “Testing Hypotheses in Evolutionary Ecology with Imperfect Detection: Capture–Recapture Structural Equation Modeling.” Ecology 93: 248–255. 22624306 10.1890/11-0258.1

[ecy70161-bib-0009] Culina, A. , F. Adriaensen , L. D. Bailey , M. D. Burgess , A. Charmantier , E. F. Cole , T. Eeva , et al. 2021. “Connecting the Data Landscape of Long‐Term Ecological Studies: The SPI‐Birds Data Hub.” Journal of Animal Ecology 90: 2147–2160. 33205462 10.1111/1365-2656.13388 PMC8518542

[ecy70161-bib-0010] Ergon, T. , Ø. Borgan , C. R. Nater , and Y. Vindenes . 2018. “The Utility of Mortality Hazard Rates in Population Analyses.” Methods in Ecology and Evolution 9: 2046–2056.

[ecy70161-bib-0011] Fay, R. , M. Authier , S. Hamel , S. Jenouvrier , M. van de Pol , E. Cam , J.‐M. Gaillard , et al. 2022. “Quantifying Fixed Individual Heterogeneity in Demographic Parameters: Performance of Correlated Random Effects for Bernoulli Variables.” Methods in Ecology and Evolution 13: 91–104.

[ecy70161-bib-0012] Fay, R. , C. Barbraud , K. Delord , and H. Weimerskirch . 2018. “From Early Life to Senescence: Individual Heterogeneity in a Long‐Lived Seabird.” Ecological Monographs 88: 60–73. 30122788 10.1002/ecm.1275 PMC6084314

[ecy70161-bib-0013] Fay, R. , S. Hamel , M. van de Pol , J.‐M. Gaillaird , N. G. Yoccoz , P. Acker , M. Authier , et al. 2022. “Temporal Correlations among Demographic Parameters Are Ubiquitous but Highly Variable across Species.” Ecology Letters 25: 1640–1654. 35610546 10.1111/ele.14026 PMC9323452

[ecy70161-bib-0014] Fay, R. , P.‐A. Ravussin , D. Arrigo , J. A. von Rönn , and M. Schaub . 2021. “Age‐Specific Reproduction in Female Pied Flycatchers: Evidence for Asynchronous Aging.” Oecologia 196: 1–12. 34173894 10.1007/s00442-021-04963-2 PMC8292251

[ecy70161-bib-0015] Forsythe, A. B. , T. Day , and W. A. Nelson . 2021. “Demystifying Individual Heterogeneity.” Ecology Letters 24: 2282–2297. 34288328 10.1111/ele.13843

[ecy70161-bib-0016] Frauendorf, M. , A. M. Allen , S. Verhulst , E. Jongejans , B. J. Ens , H.‐J. van der Kolk , H. de Kroon , J. Nienhuis , and M. van de Pol . 2021. “Conceptualizing and Quantifying Body Condition Using Structural Equation Modelling: A User Guide.” Journal of Animal Ecology 90: 2478–2496. 34437709 10.1111/1365-2656.13578 PMC9291099

[ecy70161-bib-0017] Gimenez, O. , E. Cam , and J.‐M. Gaillard . 2018. “Individual Heterogeneity and Capture–Recapture Models: What, why and how?” Oikos 127: 664–686.

[ecy70161-bib-0018] Grace, J. B. , T. M. Anderson , H. Olff , and S. M. Scheiner . 2010. “On the Specification of Structural Equation Models for Ecological Systems.” Ecological Monographs 80: 67–87.

[ecy70161-bib-0019] Hamel, S. , J.‐M. Gaillard , M. Douhard , M. Festa‐Bianchet , F. Pelletier , and N. G. Yoccoz . 2018. “Quantifying Individual Heterogeneity and its Influence on Life‐History Trajectories: Different Methods for Different Questions and Contexts.” Oikos 127: 687–704.

[ecy70161-bib-0020] Kellner, K. 2016. “jagsUI: A Wrapper Around ‘rjags’ to Streamline ‘JAGS’ Analyses.” Version 1.5.0

[ecy70161-bib-0021] Kéry, M. , and M. Schaub . 2012. Bayesian Population Analysis Using WinBUGS: A Hierarchical Perspective. London: Academic Press.

[ecy70161-bib-0022] Link, W. A. , E. G. Cooch , and E. Cam . 2002. “Model‐Based Estimation of Individual Fitness.” Journal of Applied Statistics 29: 207–224.

[ecy70161-bib-0023] Marzolin, G. , A. Charmantier , and O. Gimenez . 2011. “Frailty in State‐Space Models: Application to Actuarial Senescence in the Dipper.” Ecology 92: 562–567. 21608464 10.1890/10-0306.1

[ecy70161-bib-0024] Monaghan, P. , A. Charmantier , D. H. Nussey , and R. E. Ricklefs . 2008. “The Evolutionary Ecology of Senescence.” Functional Ecology 22: 371–378.

[ecy70161-bib-0025] Newton, I. , ed. 1989. Lifetime Reproduction in Birds. London, UK: Academic Press.

[ecy70161-bib-0026] Newton, I. , and P. Rothery . 1997. “Senescence and Reproductive Value in Sparrowhawks.” Ecology 78: 1000–1008.

[ecy70161-bib-0027] Nussey, D. , T. Coulson , M. Festa‐Bianchet , and J.‐M. Gaillard . 2008. “Measuring Senescence in Wild Animal Populations: Towards a Longitudinal Approach.” Functional Ecology 22: 393–406.

[ecy70161-bib-0028] Pearl, J. 2000. Causality: Models, Reasoning, and Inference. Cambridge, UK: Cambridge University Press.

[ecy70161-bib-0029] Plummer, M. 2003. “JAGS: A Program for Analysis of Bayesian Graphical Models Using Gibbs Sampling.” In Proceedings of the 3rd International Workshop on Distributed Statistical Computing, Vol. 124. Vienna.

[ecy70161-bib-0044] R Core Team . 2020. R: A Language and Environment for Statistical Computing. Vienna: R Foundation for Statistical Computing.

[ecy70161-bib-0030] R Core Team . 2024. R: A Language and Environment for Statistical Computing. Vienna: R Foundation for Statistical Computing. https://www.R-project.org/.

[ecy70161-bib-0031] Riecke, T. , R. Fay , H. Hegelbach , P.‐A. Ravussin , D. Arrigo , and M. Schaub . 2025. “Data from: Estimating Latent Individual Demographic Heterogeneity Using Structural Equation Models,” Dryad. 10.5061/dryad.jm63xsjp5 PMC12326905 40767375

[ecy70161-bib-0032] Riecke, T. V. , J. Hegelbach , and M. Schaub . 2023. “Reproductive Senescence and Mating Tactic Interact and Conflict to Drive Reproductive Success in a Passerine.” Journal of Animal Ecology 92: 838–849. 36708046 10.1111/1365-2656.13893

[ecy70161-bib-0033] Riecke, T. V. , B. S. Sedinger , P. J. Williams , A. G. Leach , and J. S. Sedinger . 2019. “Estimating Correlations among Demographic Parameters in Population Models.” Ecology and Evolution 9: 13521–13531. 31871663 10.1002/ece3.5809 PMC6912887

[ecy70161-bib-0034] Salguero‐Gómez, R. , O. R. Jones , C. R. Archer , C. Bein , H. de Buhr , C. Farack , F. Gottschalk , et al. 2016. “COMADRE: A Global Data Base of Animal Demography.” Journal of Animal Ecology 85: 371–384. 26814420 10.1111/1365-2656.12482 PMC4819704

[ecy70161-bib-0035] Schaub, M. , and M. Kéry . 2021. Integrated Population Models. Theory and Ecological Applications with R and JAGS. London: Academic Press.

[ecy70161-bib-0036] Stan Development Team . 2024. “Stan Reference Manual.” https://mc-stan.org/. Version 2.35.0.

[ecy70161-bib-0037] Stan Development Team . 2025. “RStan: The R Interface to Stan.” R Package Version 2.32.7. https://mc-stan.org/.

[ecy70161-bib-0038] Stearns, S. C. 1992. The Evolution of Life Histories. Oxford, UK: Oxford University Press.

[ecy70161-bib-0039] Vaupel, J. W. , K. G. Manton , and E. Stallard . 1979. “The Impact of Heterogeneity in Individual Frailty on the Dynamics of Mortality.” Demography 16: 439–454. 510638

[ecy70161-bib-0040] Vaupel, J. W. , and A. I. Yashin . 1985. “Heterogeneity's Ruses: Some Surprising Effects of Selection on Population Dynamics.” The American Statistician 39: 176–185. 12267300

[ecy70161-bib-0041] Vedder, O. , and S. Bouwhuis . 2018. “Heterogeneity in Individual Quality in Birds: Overall Patterns and Insights from a Study on Common Terns.” Oikos 127: 719–727.

[ecy70161-bib-0042] Vehtari, A. , A. Gelman , D. Simpson , B. Carpenter , and P.‐C. Bürkner . 2021. “Rank‐Normalization, Folding, and Localization: An Improved $\hat{R}$ for Assessing Convergence of MCMC (with Discussion).” Bayesian Analysis 16: 667–718.

[ecy70161-bib-0043] Wilson, A. J. , and D. H. Nussey . 2010. “What Is Individual Quality? An Evolutionary Perspective.” Trends in Ecology & Evolution 25: 207–214. 19897275 10.1016/j.tree.2009.10.002

